# COVID-19 crisis effect on HIV service delivery in Egypt: Hard times or blessings in disguise?

**DOI:** 10.4102/sajhivmed.v21i1.1170

**Published:** 2020-12-09

**Authors:** Rahma Mohamed, Heba Wanis, Sonia Zebachi, Menna-t-allah El-Kotamy, Gamal Esmat, Ahmed Cordie

**Affiliations:** 1 Endemic Medicine Department, Kasr Alainy School of Medicine, Cairo University Hospitals, Cairo, Egypt; 2 Third World Network, Cairo, Egypt; 3 Clinical Epidemiology and Aging Team (CEpiA), Mondor Institute for Biomedical Research (INSERM U955), Créteil, France; 4 Public Health Services, Henri-Mondor Hospital, Assistance Publique-Hôpitaux de Paris, Paris-Est Créteil University, Créteil, France; 5 Egyptian Patent Office, Academy of Scientific Research and Technology, Cairo, Egypt

Coronavirus disease 2019 (COVID-19) continues to disrupt the health system globally. Nevertheless, such adversity has urged us to rethink the way healthcare is delivered, based on a recommendation and a lesson learnt which we wish to highlight in the following paragraphs.

In our previous editorial published on 30 July 2020, we recommended the use of locally manufactured tenofovir disoproxil fumarate (TDF)/lamivudine (3TC) for the treatment of people living with HIV (PLHIV) in Egypt, as an alternative to the imported originator’s TDF/emtricitabine (TDF/FTC) 2-in-1 combination, the importation of which was hindered by COVID-19 restrictions, making it inaccessible.^1^

Available data about the interchangeability between TDF/3TC and TDF/FTC are not limited to their use for treatment only, but include their use for prevention as well. Evidence shows similar distribution of 3TC and FTC in cervicovaginal fluid and in semen, with a lack of data on 3TC rectal concentration in humans. In the meantime, the human mucosal pharmacokinetic profile suggests pharmacological equivalence of FTC and 3TC for pre-exposure prophylaxis (PrEP).^2^

In 2015, the World Health Organization (WHO) recommended that any person at substantial risk of human immunodeficiency virus (HIV) should be offered oral PrEP containing TDF as part of a combination HIV prevention programme.^3^ In 2017, the WHO Essential Medicines List was updated to include PrEP drugs, specifically TDF, TDF/FTC and TDF/3TC.^4^

In most countries that have adopted PrEP, TDF/FTC is the most commonly recommended PrEP therapy. Six countries recommend TDF/3TC for PrEP in addition to TDF/FTC, namely, Kenya, Namibia, Pakistan, South Sudan, Zambia and Zimbabwe, whilst Lesotho’s guidelines recommend exclusively TDF/3TC.^5^

Pre-exposure prophylaxis is a ‘game changer’ for HIV prevention. When taken consistently and correctly, it is very effective and reduces the chances of HIV infection to near-zero.^6^ Despite this, the cost of PrEP remains an important limiting factor to its wide use, particularly in low- and middle-income countries.

Egypt has the fastest growing HIV rate in the Middle East and North Africa region, where the epidemic is concentrated in key populations. Case load has increased annually by 25% – 35% for the past 10 years, and yet Egypt has not adopted the WHO’s oral PrEP recommendations at this time.^7^ Both TDF and 3TC are locally manufactured in Egypt and are available at affordable prices because of the absence of patent protection on either of them.

The growing evidence on TDF/3TC as an effective PrEP option, availability of locally produced generic TDF/3TC and the urgent need to control the trajectory of the HIV epidemic should all motivate the Egyptian National AIDS Programme (NAP) to start providing PrEP services and to recommend its use for prevention within the national guidelines. Such a step will encourage more generic pharmaceutical companies to join the growing market, increasing competition and thus further lowering costs.

During the COVID-19 pandemic, as a mitigation measure to patients’ movement restriction, the NAP implemented multi-month dispensing (MMD) of antiretroviral therapy (ART) covering 2–3 months, which has had a positive impact on the adherence of PLHIV.

In a pilot study conducted on 40 Egyptian patients attending one of the clinics affiliated to Kasr Al-Aini HIV and Viral Hepatitis Fighting Group^1^, the six-item Morisky Medication Adherence Scale^8^ was administered twice using a standard telephone script, first in mid-March and then in mid-August. The number of PLHIV with high motivation and high knowledge was significantly higher in mid-August (36 vs. 27 and 36 vs. 28, respectively, *p* < 0.001). Multi-month dispensing because of COVID-19 pandemic was the most commonly reported factor (90%) amongst those with high motivation.

It would be fair to conclude that it is necessary to move towards MMD for medically stable patients on ART, whilst managing the stock and supply line, in order to save healthcare service costs and improve patients’ adherence and retention in care.

Multi-month dispensing can be provided through community-based models of care that require the engagement of community health workers, common in rural parts of Egypt, who provide peer support to patients and help improve their adherence, retention and viral suppression.

Besides, the COVID-19 pandemic has accelerated the implementation of telemedicine in many countries, but not so much in Egypt.^9^ The use of telemedicine services for HIV prevention and management represents an innovative and a possibly more effective way of providing HIV services and for implementation of MMD in Egypt.

Whilst COVID-19 has placed pressures on our healthcare system, it has been a blessing in disguise. The above lessons learnt can certainly guide us into reshaping our healthcare system by endorsing more resource-savvy and patient-oriented strategies.

## Footnotes

1 Kasr Al-Aini HIV and Viral Hepatitis Fighting Group is a group of clinical leads in the field of infectious diseases at Cairo University Hospitals, working in collaboration with different Egyptian universities to provide high-quality care to people living with HIV and viral hepatitis, with a special focus on continuous medical education and research.

## References

[1] Cordie A, El-Kotamy M, Esmat G Antiretroviral therapy optimisation in the time of COVID-19: Is it really different in North and South Africa? S Afr J HIV Med. 2020;21(1):a1118 10.4102/sajhivmed.v21i1.1118 PMC7433226 32832117

[2] World Health Organisation Appropriate medicines: Options for pre-exposure prophylaxis [homepage on the Internet]. Geneva: World Health Organisation; 2018 [cited 2020 Aug 20]. Licence: CC BY-NC-SA 3.0 IGO. Available from: https://www.who.int/hiv/pub/journal_articles/3tc-ftc-interchangeable/en/

[3] World Health Organization (WHO) Policy brief: WHO expands recommendation on oral pre-exposure prophylaxis of HIV infection (PrEP) [homepage on the Internet]. Geneva, Switzerland: World Health Organisation; 2015 [cited 2020 Sept 5]. Available from: https://apps.who.int/iris/bitstream/handle/10665/197906/WHO_HIV_2015.48_eng.pdf

[4] World Health Organization WHO model list of essential medicines: 20th list (March 2017) [homepage on the Internet]. Geneva: World Health Organization; 2017 [cited 2020 Jul 13]. Available from: http://apps.who.int/iris/bitstream/handle/10665/273826/EML-20-eng.pdf?ua=1

[5] Hodges-Mameletzis I, Dalal S, Msimanga-Radebe B, Rodolph M, Baggaley R Going global: The adoption of the World Health Organization’s enabling recommendation on oral pre-exposure prophylaxis for HIV. Sex Health. 2018;15(6):489–500. 10.1071/SH18125 30496718

[6] Fonner VA, Dalglish SL, Kennedy CE, et al Effectiveness and safety of oral HIV preexposure prophylaxis for all populations. AIDS (London, England). 2016;30(12):1973 10.1097/QAD.0000000000001145 PMC4949005 27149090

[7] UNAIDS Miles to go – Closing gaps, breaking barriers, righting injustices: Global AIDS update 2018 [homepage on the Internet]. Geneva: Joint United Nations Programme on HIV/AIDS; 2018 [cited 2020 Sept 05]. p. 233 Available from: https://www.unaids.org/en/resources/documents/2018/global-aids-update

[8] Vural B, Acar OT, Topsever P, Filiz TM Reliability and validity of Turkish version of modified Morisky scale. J Turk Fam Phys. 1999;3(4):17–20.

[9] Khorshid M, Bakheet N, Abdallah S, Essam M, Cordie A COVID-19: A strong call for remote medicine in IBD. J Dig Dis. 2020;21(10):597–599. 10.1111/1751-2980.12935 32888261

